# Expermental investigation on adsorption of methylene blue dye from waste water using corncob cellulose-based hydrogel

**DOI:** 10.1038/s41598-024-54511-0

**Published:** 2024-02-24

**Authors:** Samuel Latebo Majamo, Temesgen Abeto Amibo, Dereje Tadesse Mekonnen

**Affiliations:** 1 Department of Chemical Engineering, College of Engineering and Technology, Wachemo University, Hossana, Ethiopia; 2 Department of Process Engineering and Chemical Technology, Faculty of Chemistry, Gdansk University of Technology, Narutowicza 11/12, 80-233 Gdansk, Poland; 3 School of Chemical Engineering, Jimma Institute of Technology, Jimma University, P.O. Box-378, Jimma, Ethiopia

**Keywords:** Corncob cellulose, Hydrogel, Methylene blue dye, Adsorption, Isotherm, Kinetics, Thermodynamics, Environmental sciences, Engineering

## Abstract

Hydrogel from corncob cellulose was synthesized in this investigation. The synthesized Hydrogel was characterized by SEM, XRD, and FTIR instruments. As the results indicate the synthesized hydrogel has required and important features, these suggest the suitability of hydrogel for the adsorption of methylene blue dye (MBD). Three important process variables (dosage, contact time, and initial concentration) with three levels were studied during the adsorption process at 30 °C and neutral pH. The efficiency of hydrogel for adsorption of MBD was determined in each experiment. The experimental results were statistically analyzed and interpreted. The maximum removal efficiency was achieved at 2.22 g/L of dosage, 80.36 min of contact time, and 74.54 mg/L of initial concentration. At this condition, 98.25% of MBD was achieved through experimental tests. Kinetics, isotherm, and thermodynamics studies were performed. Langmuir isotherm is more suitable to describe the adsorption process and the Pseudo second-order kinetic model fits this process. From the thermodynamics studies, all negative values of change in Gibbs free energy (ΔG°), and positive value of change in enthalpy (ΔH°), and change in entropy (ΔS°) indicate that the carried out experimental process is a spontaneous and endothermic. Moreover, the regeneration experiment for adsorbent was performed. The treatment of real textile industry waste water was conducted and the removal efficiency of hydrogel was 64.76%. This removal percentage reduction from sythetic aqueous solution is due to involvement of other pollutants in the real waste water. The synthesized hydrogel adsorbent is suitable up to the third cycle without significant loss in removal efficiency.

## Introduction

Nowdays, most researches are conducting to sustaine the climate^1^. Water contamination is one of the global community's current problems. It's the most widespread problem that needs research to find a solution and to use for various appropriate applications^2,3^. One widespread worldwide environmental issue that several countries face is the contamination of water from the textile industry^4^. Most organic dyes utilized throughout the textile and other industries are regarded as water contaminants. These dyes need to be removed before discharge due to their hazardous and carcinogenic features. These dyes can cause problems to aquatic animals. In addition, contaminated water by organic dye can prevent the transmission of light through the water. This directly can slow down photosynthesis. To remove dye from contaminated water, various methods are used. Biological, chemical, and physical techniques are the most common. Ion exchange, flocculation, membrane separation, adsorption using various types of adsorbents, and other ways are commonly used physical methods to remove dye from contaminated water^5,6^.

Some approaches considered as chemical methods to remove dye are UV radiation, ionization, electrochemical techniques, treatment by hydrogen peroxide, etc. Whereas as in biological approaches, microbial biomass like fungi and bacteria can be utilized as adsorbents through the adsorption process. Even though many treatments have been used, most of each has its limitations concerning environmental remediation, cost-effectiveness, and suitability for removing dyes^7,8^. The suitable dye-removing techniques should be environmentally friendly, inexpensive, and possible to utilize on a large scale. Because excess chemicals are used in the stages of processing for dye removal, these result in the generation of secondary side products to the environment. In addition, chemical treatment technique is expensive when looked at by others. The biological way of treatment is not commonly used because of its difficult procedure and challenge in applying it to remove dye^9^.

With a substantial surface area that allows for adsorption, the capacity to remove a variety of compounds quickly, and due to its low cost, the technique called adsorption is the most important and practical of the dye removal methods. Activated carbon has been a commonly used adsorbent in adsorption techniques in different industries for the last few years^10^. However, due to its expense, energy consumption, and insufficient effectiveness in eliminating pollutants, it is not recommended at the current time. In recent times, environmental bio-sorbents synthesized from biomass are more recommended for the adsorption of dye. This is due to the biocompatibility, economic feasibility, availability, and high efficiency in the removal process^11,12^.

Currently, hydrogel adsorbent is mostly recommended by indifferent researchers due to its ability to adsorb large quantities of contaminants (dye). This was its structural nature having three three-dimensional matrices containing gel that makes it more suitable to adsorb the adsorbate material in its all three-dimensional side^13^. Esterification, ionization (ion exchange), free polymerization, and cross-linking are the primary methods used for producing hydrogel. Cross-linking agents are important because they improve the hydrogel's mechanical characteristics and equilibrium swelling extent, which allows the agent's nature to change the adsorbent's properties. The hydrogel molecule's ability to retain water is due to the hydrophilic group that is attached to the matrix of polymers. Cation dye removal from wastewater has historically been difficult and has become a common issue. Nevertheless, hydrogel has proven to be a highly effective material for dye adsorption, exhibiting an exceptionally high level of methylene blue dye adsorption^14^.

The area of research that is currently needed is coming up with suitable raw materials for hydrogel synthesis and looking into their suitability for dye removal by performing a detailed investigation of kinetics, isotherm, and thermodynamics studies. That's why hydrogel made of biomass cellulose is receiving particular attention because of its biocompatibility, biodegradability, renewability, and availability. In addition, other benefits are low cost, low environmental impact, and adsorption of high quantities of dye. Linear cellulose chains are straight and support the formation of crystalline and morphological structures because cellulose can form intra- and between-molecular hydrogen bonds through its interaction of hydroxyl groups within it^15^.

Most literatures used cellulosed based hydrogel for waste water treatment including removal of methylene blue dye. However, there is research gap in perspective of optimization of process parameters, full analysis of kinetics, thermodynamics and isotherm of expermental investigation. In addition, most related research conducted by variable pH value. The pH of neutral is highly recommended in most science. By looking these research gap, the current study interested to perform investigation on these behalves. The study performed cellulose extraction, characterization, hydrogel synthesis, hydrogel characterization, and evaluation of hydrogel efficiency in MBD removal at neutral pH. For the adsorption of MBD, 20 experiments were designed using the central composite design method (CCD) of design expert version 11 software. Moreover, kinetic studies, isotherm, and thermodynamics of the adsorption process were performed in this study.

## Materials and methods

### Chemicals and reagents

In this study, high-quality chemicals were employed in the experiments. The principal chemical substances used in this investigation are high-grade methylene blue dye, citric acid (C_6_H_8_O_7_, Mumbai, India, 99.5% extra pure), polyethylene glycol (England), H_2_SO_4_ (99.78% pure), NaOH (98% pure), H_2_O_2_ (50% pure), ethanol (97% pure), and phosphoric acid (H_3_PO_4_, Mumbai, India). All of the solutions were made with distilled water.

### Cellulose extraction from corncob

The sample of corncobs was cleaned three times with distilled water in order to get eliminate of any impurities. It was then grinded into fine powder and dried in an oven at 80 °C for two hours. The dried fine powder of corncob sample was subjected to three stages of extraction, following standard procedures suggested in literature^16^. In order to extract cellulose from corncob for this investigation, the optimum extraction conditions recommended in the literature^16^ were used.

#### Stage I: Alkaline pretreatment

In the first stage, preatreatment of sample with base was carriedout in a 2L volumetric falsk having 10% NaOH solutions of 1:10(w/v) of solid corncob sample to liquid (water) ratio. After that, the mixture was then heated at 90 °C for 1.5 h in a water bath. Preheated distilled water was used to remove the residue, and sample was frequently filtered.

#### Stage II: Hydrogen peroxide, acetic acid and formic acid treatment

The sample obtained from first satge was proceeded to further treatment with a solution containing 7.5% H_2_O_2_, 20% acetic acid, and 20%formic acid. After that, it was placed in a water bath and run for 1.5 h at 90 °C. Then, it proceeded to cool for half an hour at room temperature. A vacuum filter was subsequently utilized to separate the cooking liquor (hemicellulose and lignin) from cellulose. After that, it undergone continuous washings in hot distilled water until the pH reached neutral.

#### Stage III: Bleaching

The obtained sample from stage II was bleached in basic media containing 4% NaOH and treated with 7.5% H_2_O_2_. It was then placed in a water bath and stirred using a magnetic stirrer at 70 °C for 30 min. The pulp was then repeatedly rinsed in hot distilled water to remove any remaining lignin, and it was then dried in an oven at 60 °C until a constant weight was achieved.

### Hydrogel synthesis

The process for producing corncob-cellulose-based hydrogel was carried out by following the procedure of previously published literature with minor modifications^17^. 5 g of cellulose sample was added into the beaker, then 20 mL of concentrated H_3_PO_4_ to break down cross-linked and long-chained cellulose structure by means of dissolution of cellulose. For 24 h, the cellulose and H_3_PO_4_ mixture was stirred continuously with a magnetic stirrer until the mixture completely dissolved at room temperature. After that, a gram (w/w) of polyethylene glycol was added to each of the cellulose mixtures, and the mixtures were mechanically stirred for 30 min to achieve a homogenous solution. The mixtures were then mixed with 15 mL of a 10% (w/w) citric acid solution, which was stirred for 30 min at room temperature using a magnetic stirrer. The completed solution is then stored at room temperature without a string for a full day to ensure complete homogeneity. At last, the solution was heated up at 50 °C after being poured in a 40 mm sized petri dish. Synthesized hydrogel proceeded for characterization.

### Characterization of adsorbents

The bonds and groups available on the surface of adsorbents were determined by using Fourier transform infrared spectroscopy (FTIR, L1600300 Spectrum TWO UTA). Cellulose and hydrogel samples’ spectrum was evaluated in between 4000 and 400 cm^−1^ of wavelength after being distributed in a KBr matrix at a concentration of 5 mg per sample. Scanning electron microscopy, SEM (Model: FEI INSPECT F50, Japan) was used to examine the morphology and structures of the adsorbents, while the X-ray diffractometer (XRD-7000) was used to analyze the crystallographic and amorphous properties of the adsorbent materials that operated at voltage of acceleration of 30 kV and current of 25 mA, and the UV–Visible spectrophotometer (6100Z, Jenway) was used to examine the dye content before and after adsorption.

### Adsorption experiment

The procedure of batch adsorption was used to remove MBD from an artificial liquid solution. The batch tests have been set up by varying the initial MBD content. The laboratory tests conducted in batch mode aimed to evaluate the impact of varying amounts of adsorbent (2–8 g/L), time of contact (30–150 min), and the initial concentration of dye (50–200 mg/L) on the hydrogel removal efficiency. Every adsorption test was run at 30 ± 1 °C and neutral pH (pH = 7). The operations were carried out in a shaker running at 200 rpm and 30 ± 1 °C. Ultimately, water and adsorbent leftovers were separated from mixtures by filtering them through watchman filter paper. The MBD quantities were measured using a spectrophotometer at a maximum wavelength of 665 nm. As indicated in Table 1, three factors with three levels were designed using CCD of design expert version 11 software.

**Table 1:** Factors and labels for experiment design.

Factors	labels		
	Low	medium	high
Adsorbent dose (g/L)	1	2	3
Initial dye concentration (%)	50	100	150
Contact time (min)	40	70	100

The numbers of required experiments for three key factors were computed using Eq. (1)

1 $$N = 2^{n} + 2n + n_{c\,}$$

where N is the total number of conducted experiments, n is the number of key parameters (selected factors), and n_c_ is the number of points at the center (6 replications). A total of 20 tests were conducted. The response parameter (MBD removal efficiency) was correlated by design expert software using three selected factors as shown in Eq. (2)

2 $${\text{Removal efficiency}} = {\text{b}}_{{\text{o}}} + \mathop \sum \limits_{{{\text{i = }}1}}^{3} {\text{b}}_{{\text{i}}} {\text{X}}_{{\text{i}}} + \mathop \sum \limits_{{{\text{i = }}1}}^{3} {\text{b}}_{{{\text{ii}}}} {\text{X}}_{{\text{i}}}^{2} + \mathop \sum \limits_{{\text{i}}}^{{}} \mathop \sum \limits_{ < j = 2}^{3} {\text{b}}_{{{\text{ij}}}} {\text{X}}_{{\text{i}}} {\text{X}}_{{\text{j}}} \, + e_{i} \,$$

where; X_i_ and X_j_ refer to variables (i and j range from 1 to k), b_o_ stands for intercept at center; b_j_, b_jj_, and b_ij_ are coefficients of interaction of variables; and e_i_ is the error. Removal efficiency of hydrogel was determined according to Eq. (3).

3 $${\text{Re}} moval\,efficiency\,\,(\% ) = \frac{{C_{o} - C_{e} }}{{C_{o} }}*100$$

where; C_o_ and C_e_ are the final and initial concentrations of MBD (mg/L).

Adsorption capacity of adsorbent qe (mg/g) was computed using Eq. (4)

4 $$Adorbent\,adsorption\,capacity\,(mg/g) = \frac{{C_{o} - C_{t} }}{M}*V$$

where; qe, Co, Ct, M, and V were capacity of adsorption, initial mass concentration of MBD, equilibrium mass concentration of MBD, mass of adsorbent used at optimum condition, and volume of aqueous solution respectively.

### Adsorption isotherm

The isotherms for adsorption were examined in this investigation under optimal conditions. In order to identify which isotherm model (Freundlich and or Langmuir) would best explain and forecast the adsorption of dye onto hydrogel, its relevance was tested. The Langmuir isotherm model can be expressed using linear and non linear equations represent by Eq. (5) and Eq. (6) respectively, proposes that adsorption takes place on homogeneous surfaces.

5 $$\frac{{C_{e} }}{{q_{e} }} = \frac{1}{{q_{\max } *K_{L} }} + \frac{1}{{q_{\max } }}*C_{e} \,\,(Linear\,equation)\,$$

6 $$q_{e} = \frac{{q_{m} k_{L} C_{e} }}{{1 + k_{L} C_{e} }}\,\,\,\,\,({\text{non - linear}}\,{\text{equation}})$$

where C_e_/q_e_ is the main response, 1/(q_max_ * b) is the intercept and 1/q_max_ is slope. The q_e_ (mg/g) is the equilibrium adsorption capacity, q_max_ (mg/g) is the maximum adsorption capacity, C_e_ (mg/L) is the equilibrium concentration of dye, and b (1/mg) is the Langmuir adsorption constant. The important isolation factor (R_L_) was determined using Eq. (7)

7 $$R_{L} = \,\frac{1}{{\left( {1 + K_{a} C_{o} } \right)}}$$

When R_L_ = 0, the process is irreversible; R_L_ = 1, it is linear; R_L_ > 1 unfavorable; and 0 < R_L_ < 1 (favorable).

The non-ideal adsorption behavior can be described by the Freundlich isotherm model. This isotherm model explains the adsorption process in heterogeneous surfaces. The Eq. (8) and Eq. (9) is used to correlate the isotherm model.

8 $$\ln q_{e} = \ln K_{F} + \frac{1}{n}\ln Ce\,\,\,(linear\,equation)$$

9 $$q_{e} = K_{F} *(Ce)\,^{\frac{1}{n}} \,\,\,\,\,\,(non\,linear\,equation)$$

where qe capacity of adsorbent (mg/g), K_F,_ and n are the isotherm constants of the Frendlich model. 1/n used to verify the adsorption process favorability.

### Adsorption kinetics

Studying the kinetics of the adsorption process is very important to determine the suitable order of reaction. The pseudo-second and pseudo-first-order models were evaluated to select the best-describing model. The pseudo-first-order kinetic model was determined and correlated using Eq. (10) and the pseudo-second-order kinetic model was evaluated using Eq. (11).

10 $$\frac{{d_{qt} }}{dt} = K_{1} (q_{e} - q_{t} )$$

11 $$\frac{t}{qt} = \frac{1}{{K_{2} q_{e}^{2} }} + \frac{1}{{q_{e} }}\,\,(t)$$

where k_1_ (min^−1^) and K_2_ (g/mg min) are rate constants of pseudo first and second-order adsorption, q_t_ (mg/g) is the quantity of MBD adsorbed at time (min), q_e_ (mg/g) is adsorption capacity at equilibrium, t (min) is the contact time.

### Adsorption thermodynamics

Studying the thermodynamics for adsorption is very important to find out the type of process and the type of reaction carried out in the process. In this investigation changes of Gibbs free energy (ΔG°), enthalpy change (∆*H*), and entropy change (∆*S*) were evaluated. The amount of energy stored in bonds can be expressed by enthalpy and the change of system in the adsorption process can be expressed by entropy or entropy change. The ΔG° is used to decide the spontaneity of the adsorption process. These parameters were correlated using Eqs. (12, 13, 14):

12 $$\vartriangle G^{ \circ } = RT\ln K_{c} \,$$

13 $$\ln K_{c} = \frac{{\vartriangle S^{ \circ } }}{R}\, - \frac{{\vartriangle H^{ \circ } }}{RT}$$

14 $$\Delta G^{ \circ } = \,\Delta H - T\Delta S$$

where Kc (L/g) is the distribution coefficient, R (8.314 J/mol K), and T is temperature (°K).

## Results and discussion

### Adsorbents characterization

#### FTIR characterization result

**Figure 1:**
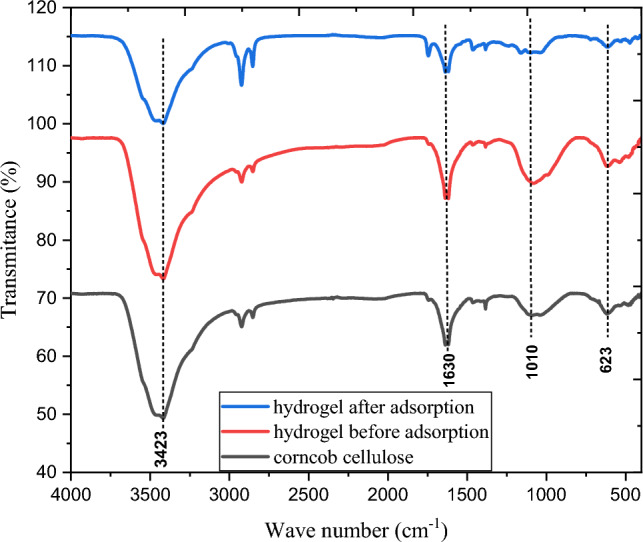
FTIR spectrum bands of corncob cellulose and corncob cellulose-based hydrogel after and before adsorption.

Figure 1 represents the bonds involved in functional groups and attached components of the corncob cellulose, hydrogel before use and hydrogel adsorbent after fourth cycle use with their corresponding FTIR spectra. The hydroPeaks at 3423, 2900, 1630, 1010, and 623 cm^−1^ were frequently observed. Some of these peaks, though, have varying intensities. In all samples, the peak at 3423 cm^−1^ is linked to O–H stretching vibration. In hydrogel sample before use has a higher intensity than in cellulose and hydrogel adsorbent after use.

When compared to the intensity of the cellulose sample, it suggests that hydrogel adsorbent before use has a more hydrophilic nature^18^. The hydrophilic character of hydrogel prevents the formation of hydrogen bonds in its intermolecular chain for water sorption^19^. The stretching movement of C–H is represented by the peak at 2900 cm^−1^ in all three samples^22^. The hydrogel's (before use) peak at 1630 cm^−1^, which has a higher intensity, shows that the esterification reaction effectively introduced the carbonyl group (C=O) into the hydrogel. The C–C and C–O cellulose skeleton motion in the hydrogels, which band at 1010 cm^−1^ rose in comparison to corncob cellulose and hydrogel adsorbent after use^23^. This suggests the existence of the ester bonds (C–O) in hydrogel adsorbent before use proving the well interconnected gels. However the low intensity of hydrogel adsorbent after use indicates that distruction of C–C and C–O ester bonds in adsorption media due to applying mechanical energy (strring of the sample) in each cycle and through continuous washing. A comparable maximal in cellulose has been associated with the aliphatic C-H bonds of cellulose, hemicellulose, and lignin^24^. The decline in cellulose intensity in the 623 cm^−1^ region can be linked treatment of cellulose with base. Specifically, the point suggests that the hydrogels before use have undergone a crystal change from cellulose I to cellulose II. Overall, hydrogel exhibited the different spectrums that indicate the interconnected networks in its matrix^25^. The highly decline in hydrogel adsorbent after use intensity in the 623 cm^−1^ indicates that loss of crystalline nature through continuous treatment after each cycle upto four run.

#### XRD analysis

**Figure 2:**
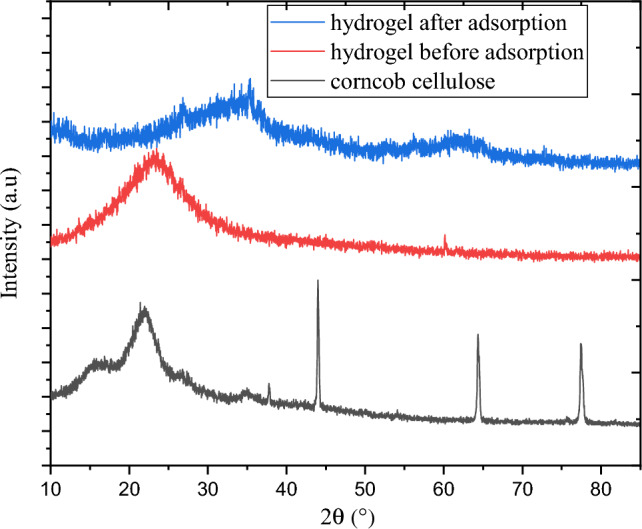
XRD diffraction pattern of corncob cellulose and corncob cellulose-based hydrogel after and before adsorption.

The XRD results of the hydrogel synthesized from corncob cellulose before and after use, and corncob cellulose were shown in a single plot in Fig. 2. The diffraction was performed with a scanning rate of 0.03 s^−1^. There was clear change was observed in the diffraction pattern of all three samples. As illustrated in Fig. 1, cellulose exhibited crystalline peaks at 2θ of 44.00°, 64.34°, and 77.40°. These peaks represent that cellulose is highly crystalline when compared with hydrogel^18^. This was evidenced by the crystalline index of cellulose is 18.03%. There is not any crystalline sharp peak observed in the hydrogel. The reduction of crystalline peaks in hydrogel indicates that hydrogel is less crystalline. As was already indicated, the –OH group in the material was eliminated by the cross-linkage connection, resulting in an overall reduction in the bonding of hydrogen and ensuing decline in crystalline structure^23^. In generally this enhances the swelling capacity of hydrogel and the hydrogen can be a suitable adsorbent for MBD removal. Hydrogel after use has shown variation in its structural pattern from hydrogel adsorbent before use. The broad band peak around 2θ of 22.00 in hydrogel sample before use shifted to 2θ of 35.00 and its intensity also decreased. This indicates that after fourth cycle, the amorphose nature reduced via continuous treatment by washed with a solution of NaOH during regeneration of adsorbent.

#### SEM analysis

**Figure 3:**
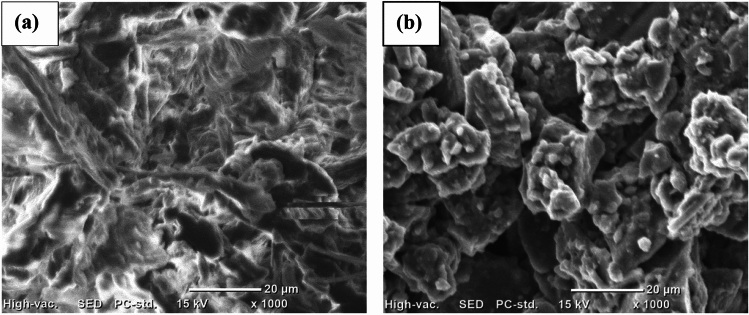
SEM image of corncob cellulose (**a**), and corncob cellulose-based hydrogel (**b**).

The morphology of corncob cellulose and hydrogel were represented in Fig. 3a,b respectively. The SEM image in Fig. 3a shows the different graded cellulose structures that are connected together. As illustrated from the images, the surface morphology of cellulose was smoother than that of hydrogel. This indicates that hydrogel is better for the adsorption process. In addition, there are very less porous holes in cellulose structure, while hydrogel is highly porous as compared to cellulose. This enables the hydrogel to have enough porous media to adsorb the MBD. Furthermore, the surface morphology of hydrogel exhibited the transparent feature, this confirms the synthesized hydrogel is pure product. The morphology of hydrogel synthesized in this study conformed to the surface morphology of hydrogel synthesized from cellulose chitosan-graft and nanofibrils^26^. When compared to cellulose morphology, is cross-linked structure has been observed in the hydrogel. This coma from the esterification reaction during synthesis of hydrogel.

### Adsorption mechanism using cellulose based hydrogel

The adsorption mechanisim of current investigation is by both chemical and physical means. The used MBD is cataionic and hydrogel synthesized via esterification in this study is anaionic adsorbent. Scince, OH^-^ groups attached on the surface of the adsorbent, hydrogel is anionic which need cataions to be stable. The negatively charged hydrogel surface provided electrostatic attraction with positively charged MBD. This favours the highest adsorption capacity of hydrogel. In addition, some portion of MBD could be adsorbed in the porous media of adsorbent particles. In this case there is also physical adsorption. The desorption of adsorbent easily carriedout using 0.1 M solution of NaOH.

### Experimental results and statistical analysis

Table 2 involved the experimental results at designed points and predicted response. The statistical validity of the quadratic model was evaluated using the ANOVA. The model terms' reliability and significance have been determined using the F-value, p-value, and lack of fit^27^. The relevance of the model terms was usually demonstrated by a variable's greater F-value and smaller P-value^28^. The quadratic model was significant, as indicated by the obtained F-value of 263.54 (p < 0.0001). ANOVA theory states that every coefficient term whose p-value is below 0.0100 denotes the statistically significant nature of the model terms^29^. The model with quadratic terms (A^2^, B^2^, and C^2^), the model with linear terms (A, B, and C), and the model with term interactions (AB, AC, and BC) were all significant according to this theory. The statistical insignificance in relation to pure error was indicated by the p-value and F-value of lack of fit, which were obtained from the ANOVA and were 0.0561 and 4.75 respectively. The model's suitability is thus indicated by a non-significant feature of lack of fit^30^.

**Table 2:** Designed experiments and corresponding response values.

Run	Dose	Time	Initial concentration	Removal efficiency	Predicted
1	2	70	100	92.28	92.06
2	2	70	100	91.92	92.06
3	3	40	150	69.18	68.10
4	3	100	150	78.28	79.05
5	3	40	50	92.33	92.70
6	3	100	50	97.61	97.65
7	2	70	100	92.87	92.06
8	1	70	100	70.31	71.34
9	3	70	100	91.91	91.81
10	2	100	100	96.47	95.42
11	2	40	100	82.37	84.35
12	2	70	50	96.15	95.90
13	2	70	150	76.52	77.70
14	1	40	150	48.18	47.91
15	1	100	150	65.71	65.11
16	1	100	50	76.06	76.91
17	1	40	50	66.71	65.71
18	2	70	100	91.28	92.06
19	2	70	100	93.31	92.06
20	2	70	100	92.53	92.06

High values for the coefficient of correlation terms, coefficient of variation, and adequate signal verified the recommended model's significance and suitability. With R^2^ value of 0.995 and an adjusted R^2^ value of 0.992 (Table 3), the experimental results' maximum variation was explained by the quadratic theory. This further indicates that the actual results from the test that was performed were more in line with the predicted results. An adequate signal is also indicated by a signal-to-noise ratio (adequate precision) of 57.990. Furthermore, the ANOVA analysis shows that the tests were conducted with better precession and validity, as evidenced by the much lower coefficient of variation value (C.V. = 1.460%) as shown in Table 3. A model can be believed quite reproducible due to its C.V. value.

**Table 3:** Summary of the adequacy of a model.

R^2^	0.995
Adjusted R^2^	0.992
Predicted R^2^	0.963
Adequate precision	57.990
Coefficient of variation	1.46%

#### Development of a model equation

The relationship between factors and the outcome variable was well-explained by the polynomial equation for the proposed model term. The establishment of a model is essential to forecasting response change, or the shift in the hydrogel's removal efficiency, within a specific operating variables range. The coded factors intercept lies at the precise center of the space of design. It is crucial to determine response value using an equation built based on the coded factor. Equation (15) represents the regression model's finalized equation in terms of coded variables.

15 $$\begin{gathered} {\text{Re}} movalefficiency(\% ) = 92.06 + 10.23*A + 5.54*B - 9.10*C \hfill \\ - 1.56*A*B - 1.70*A*C + 1.50*B*C - 10.48{*}A^{2} - 2.17{\text{*B}}^{2} - {5}.26{*}C^{2} \hfill \\ \end{gathered}$$

where A—adsorbent dose; B—contact time; C—initial adsorbate concentration.

**Figure 4:**
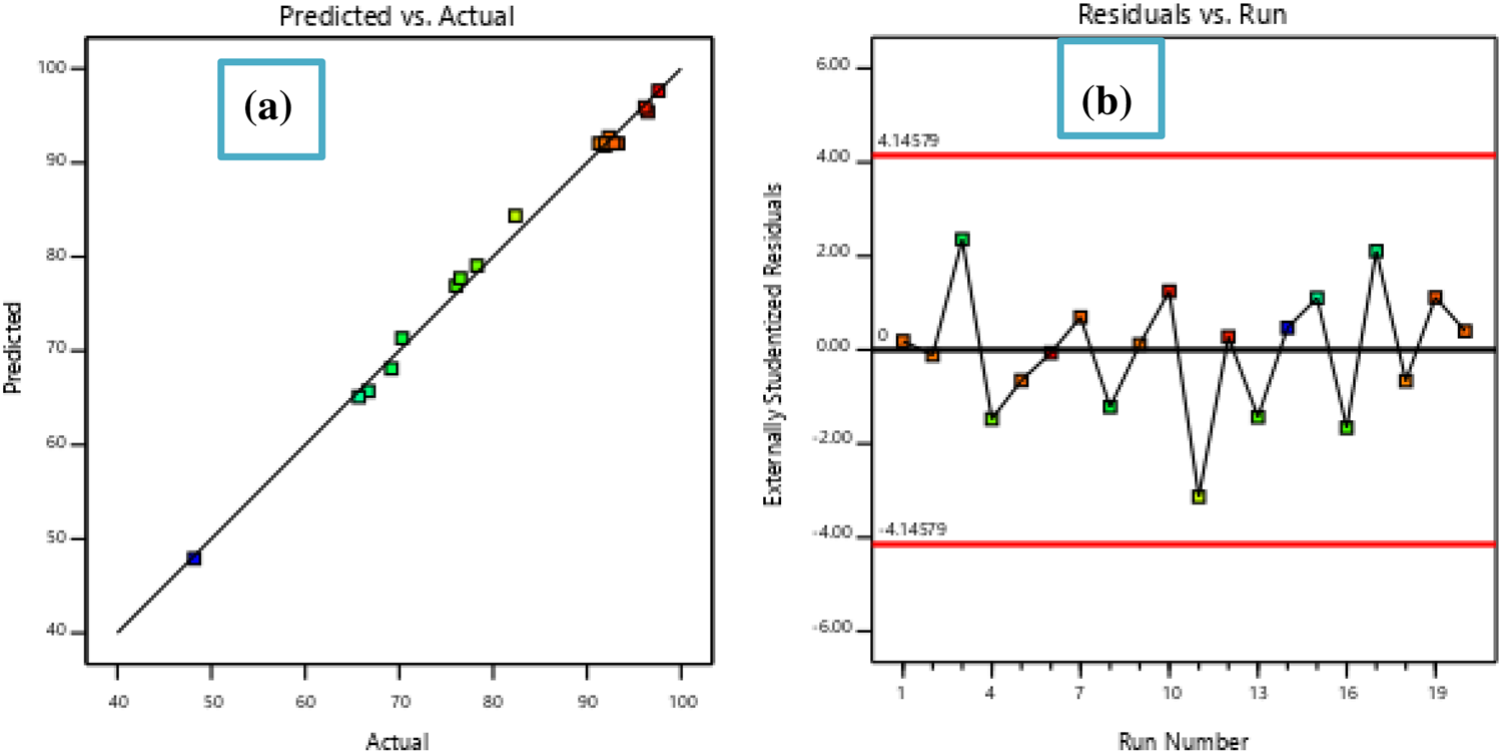
The actual versus predicted removal efficiency (**a**), the plot of residuals versus run number (**b**).

Figure 4a illustrates how the experimental values verse predicted the MBD removal efficiencies. All the values are a lined on linear straight line. The linear line drowning at 45 degrees suggests a closer relationship between the predicted and actual removal efficiencies. The high value of R^2^ = 0.995, which was closer to unity, provided additional support for it. This suggests that the experimental data agreed reasonably well with the model, indicating that the data fit the model well^29^. The plot of residuals against run number in Fig. 4b validates the significance of the specified model. It also shows a random dispersion of residuals in relation to the predicted values.

### Interaction effect of process variable in removal efficiency of hydrogel

**Figure 5:**
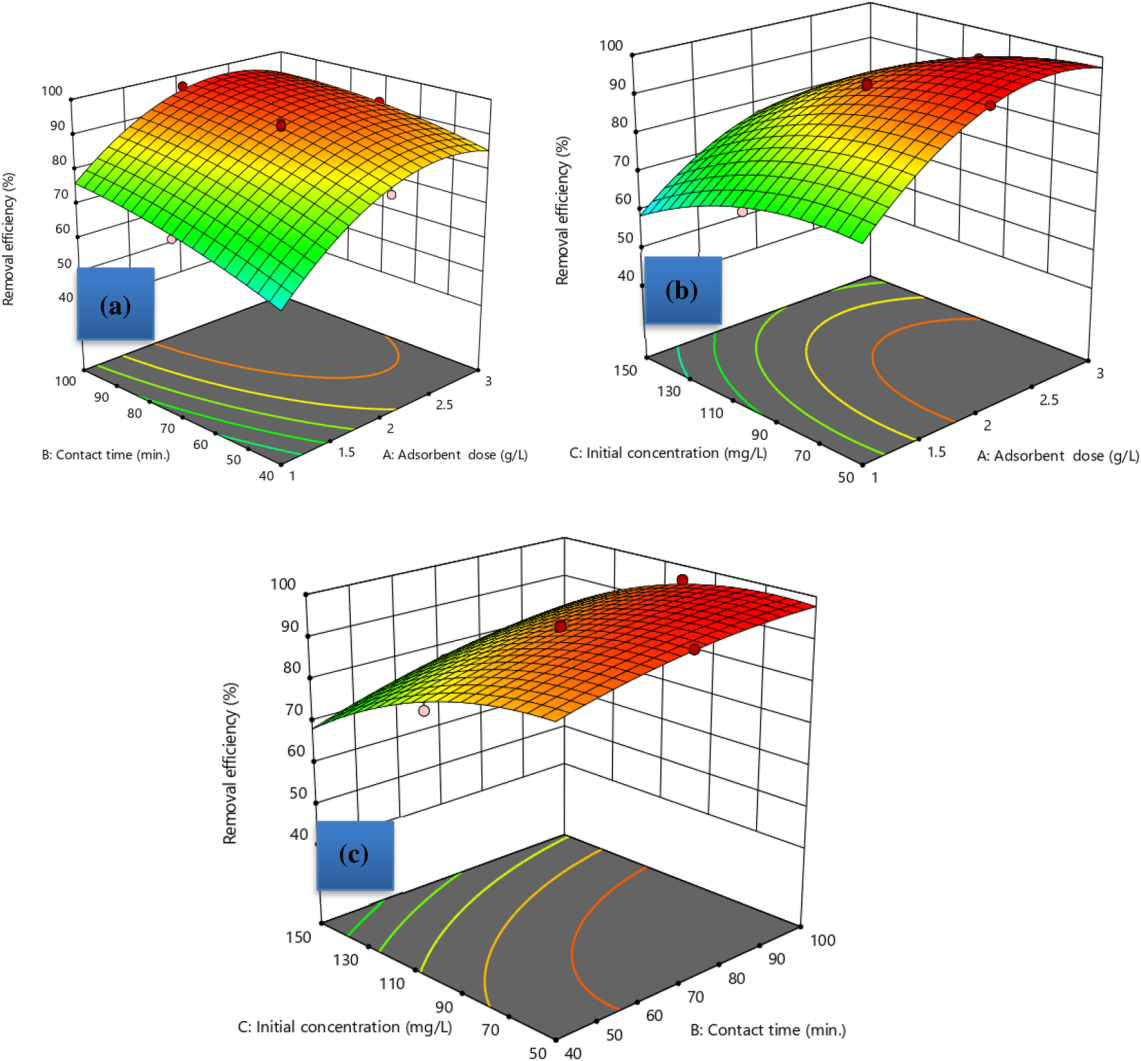
3D plots of the interaction effect of investigated variables on removal of MBD.

Corncob cellulose-based hydrogel removal efficiency was significantly impacted by the association of all three factors under investigation. The 3D surface plot in Fig. 5a illustrates the relationship that exists between the effects of adsorbent dosage to contact time. The percentage of MBD removed is low at low adsorbent doses and contact times. As an illustration, the removal efficiency was 66.72% when the contact time was 40 min and the dosage was 1 g/L with an initial concentration of 50 mg/L. This occurred because there is insufficient adsorbent used to adsorb MBD, and there is insufficient operating time for the adsorbent to make maximum contact with the adsorbate on its surface. 82.37% removal efficiency was obtained at a medium dosage (2 g/L), a low contact time (40 min), and a medium initial concentration (100 mg/L). Similarly, 70.32% removal efficiency was obtained at medium initial concentration, medium contact time (70 min), and low dosage (1 g/L). The removal efficiency was somewhat raised by increasing at least one variable (either dosage or contact time). The removal efficiency of hydrogel increased with increasing adsorbent dosage and contact time^31^. This results from an increase in the adsorption active site with adsorbent dosage increments as well as an increase in the electrostatic force between the adsorbent active site and the adsorbate with time. However, the third factor—the initial concentration—had a negative impact on MBD removal.

The interaction effect between MBD dosage and initial concentration is depicted in Fig. 5b. The removal efficiency of hydrogel increased with an increase in adsorbent dosage and a decrease in MBD initial concentration. The interaction between these two factors is statistically significant, as indicated by a lower P value (p < 0.0027). This suggests that the adsorbate is mostly adsorbed on the surface of the adsorbent. When there is little MBD in the aqueous solution, the highest dosage is more reasonable. Figure 5c displays a more comparable pattern of the relationship between contact time and MBD's initial concentration. However, in comparison to the other discussed interactions, their significance of theirs was low (P = 0.0057). Maximum contact time at low MBD concentration led to higher hydrogel removal efficiency^32^.

### Numerical optimization and model validation

Table 4 includes every considered variable including response within the ideal range. Increasing the hydrogel's efficacy for removal in the specified range of process parameters was the ultimate objective of this investigation. The composite desirability as well as the mean of three replicate tests carried out at the specified optimal state was subsequently utilized to assess the obtained best circumstances. The software generated an overall desirability of 1.0 at the suggested optimal conditions. It shows that the desired level of response has been achieved effectively in terms of the degree of fulfillment of the best possible circumstances. The suggested removal efficiency was 98.55%, whereas the mean value of the MBD removal of triplicates was 98.25%. The experimental and software results at optimal conditions proved an extremely low deviation (0.3%), indicating that the model matched the experimental findings.

**Table 4:** Optimum conditions and model validation.

Process variables	Unit	Optimum results
Adsorbent dose	g/L	2.22
Contact time	Min	80.36
Initial concentration	mg/L	74.54
Removal efficiency from software	%	98.55
Removal efficiency from the experiment	%	98.25
Deviation	%	0.30
Desirability	–	1

#### Adsorption isotherm

The adsorption capabilities of the experimental results were examined using the Freundlich and Langmuir adsorption isotherms as shown in Table 5. At 30 °C and neutral pH, the initial concentration of 74.54 mg/L, the dosage of 2.22 g/L, and the contact time of 80.36 min, the maximum removal of MBD was achieved. During the isotherm study, the initial MBD concentration was varied whereas all the parameters were kept constant. Isotherm tests at six different initial MBD concentrations were conducted. The linear and non-linear isotherm model are very important to deep analysis of isotherm model^33^. The linear and non linear isotherm equations were used in this study.

**Table 5:** Results of adsorption isotherms of MBD removal experiment.

Isotherm models	Parameters	Linear isotherm values
Langmuir isotherm model	q_m_ (mg/g)	55.31
	K_L_ (L/mg)	0.418615
	R^2^	0.994
	R_L_	0.031052
Freundlich isotherm model	1/n	0.23961
	K_F_	23.48237
	R^2^	0.969

**Figure 6:**
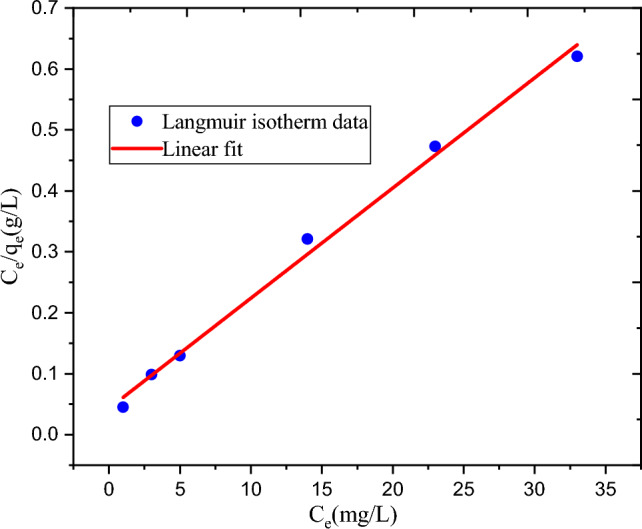
Graphical representation of Langmuir adsorption isotherm.

**Figure 7:**
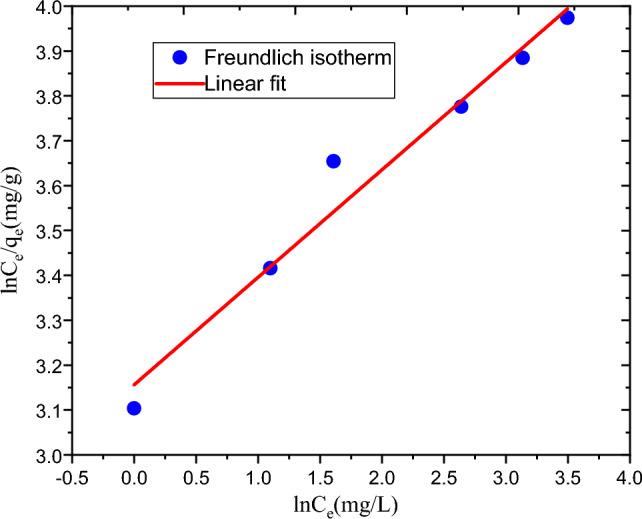
Graphical representation of Freundlich adsorption isotherm.

**Figure 8:**
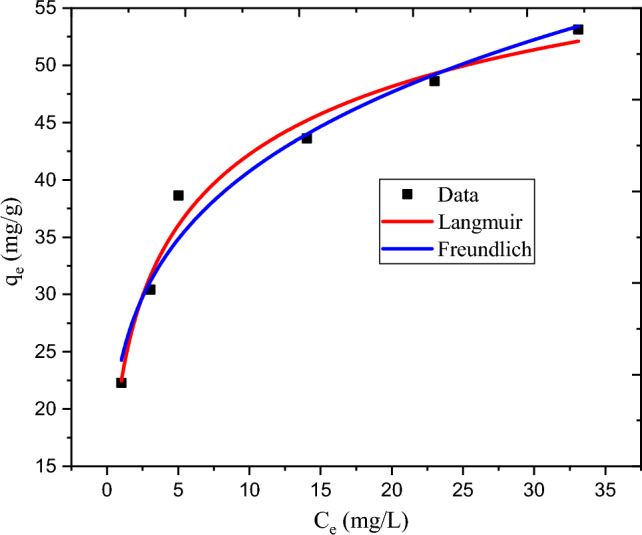
Graphical representation of non-linear Langmuir and Freundlich adsorption isotherm.

The relationship between the amount adsorbed at a constant temperature (3 °C) and neutral pH, the MBD aqueous state activity is shown by the Langmuir adsorption isotherm in Fig. 6. Furthermore, the Freundlich adsorption isotherm is shown in Fig. 7. The combined non-linear isotherm plots were shown in Fig. 8. The R^2^ values of linear and non-linear Langmuir isotherm model were 0.994 and 0.970 repectively. In addition, R^2^ values of linear and non linear Freundlich isotherm model were 0.969 and 0.964 respectively. There was greater variation in R^2^ in Langmuir isotherm, but still its coefficient of variation is higher than that of Freundlich. The best-fitting isotherm model is indicated by an isotherm model that has a lower error and a higher R^2^^34,35^. Therefore Langmuir isotherm is suggested to represent present experiment. Additionally, the 0.031 dimensionless separation factors (R_L_) value falls between 0 and 1, suggesting that the adsorption process in the current experiments is favorable. All in all, the Langmuir adsorption isotherm can be used to describe the adsorption process in the present investigation. The present experiment's highest capacity for adsorption (q_m_ = 55.31 mg/g) shows that the developed hydrogel adsorbent has a good ability for MBD adsorption.

#### Adsorption kinetics

Kinetics of adsorption were widely used to provide the best circumstances for batch processes run at full scale, as well as to explain the sorption mechanism and potential governing step^36,37^. In addition to providing complete information for developing and testing the adsorption process, the kinetic parameters studied via pseudo both first and second-order analyses serve as useful to estimate the rate of adsorption^43^. The maximum R^2^ indicated that the kinetic model was conformed and acceptable. Important results generated from the kinetics model are involved in Table 6.

**Table 6:** Kinetic models and model parameters’ values.

Kinetics models	Parameters	Values
Pseudo first order	q_e_ (mg/g)	2.483776
	K_1_ (L/mg)	− 0.01127
	R^2^	0.949
Pseudo second order	q_e_ (mg/g)	35.12469
	K_2_	0.005373
	R^2^	0.999

**Figure 9:**
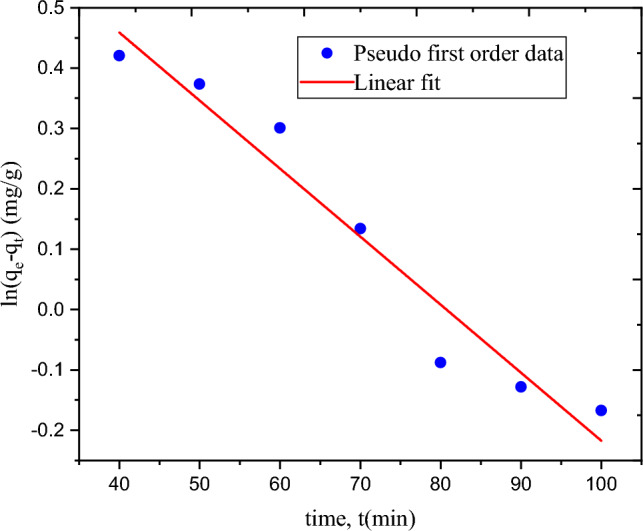
Pseudo first-order adsorption kinetics for adsorption of MBD using hydrogel.

**Figure 10:**
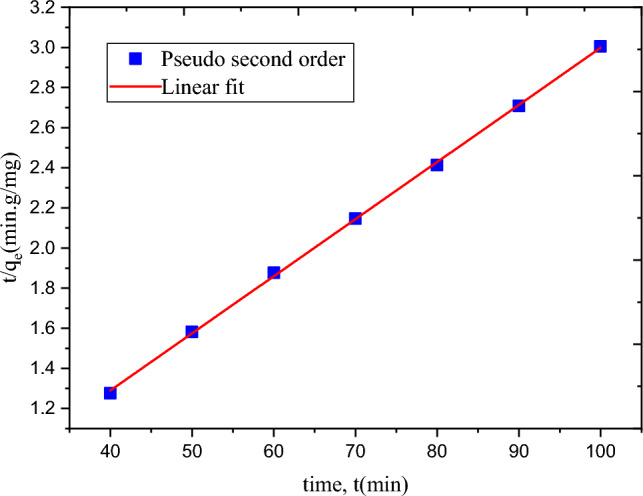
Pseudo Second order adsorption kinetics for adsorption of MBD using hydrogel.

Figure 9 shows the time, t, versus ln(qe-qt) plot for first-order kinetics. Figure 10 displays the second-order kinetic system of time, t, versus t/qe plot. The rate constants were determined for both kinetic models. The computed values of the correlation (R^2^), rate constants, and adsorption capacity for both models are listed in Table 6. R^2^ values for first- and second-order kinetic models are 0.949 and 0.999, respectively. The adsorption process fits the second-order kinetic model, according to the maximum R^2^ value for the second-order model^39^. This means that the second-order kinetic model can be used to describe the removal of MBD through adsorption using cellulose-based hydrogel.

#### Adsorption thermodynamics studies

**Figure 11:**
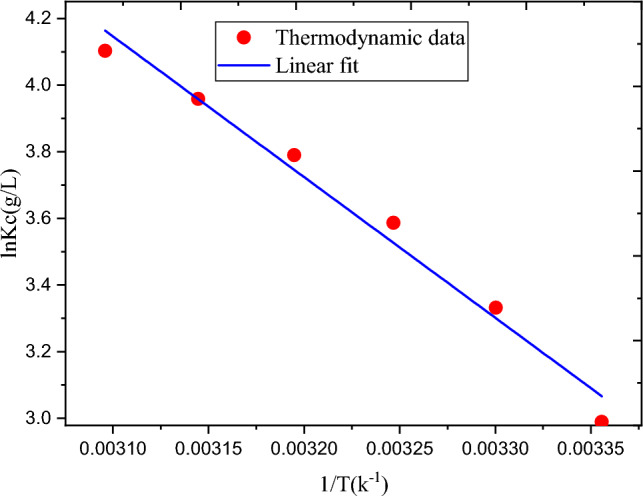
The plot of lnK_C_ verses 1/T on adsorption of MBD process using hydrogel.

One of the primary goals of thermodynamics is to provide metrics to determine whether a specific process will occur spontaneously or not^40^. The thermodynamic characteristics of MBD removal employing a corncob cellulose-based hydrogel were studied. The important thermodynamic parameters: changes of enthalpy (ΔH°), changes of entropy (ΔS°), and changes of Gibbs free energy (ΔG°) were calculated. The obtained values are available in Table 7. Figure 11 presents the plot of lnK_C_ verses 1/T. The intercept and slope were used to compute the ∆S° and ∆H° respectively. A higher R^2^ value (0.981) supported the calculated thermodynamic parameter values, indicating the linear line through the data points was fitted to the experimental data as illustrated in Fig. 11. Every ∆G° value was negative and decreased as the temperature rose, suggesting that MBD was adsorbed using hydrogel takes place spontaneously and that the reaction was feasible thermodynamically^41^. In other words, compared to products, reactants have greater free energy. The endothermic nature of the MBD adsorption process was verified by the positive ∆H° value of 35.11 kJ/mol. The ∆S° value was also positive (0.14 J/Kmol), which explains solid/solution interface is more disordered within the system during MBD adsorption by hydrogel^42^. Additional energy is not required for more disordered processes to perform adsorption^43^.

**Table 7:** Thermodynamic parameters for the adsorption of MBD at various temperatures.

Temperature, T (K°)	ΔG° (KJ/mol)	ΔH° (KJ/mol)	ΔS° (J/Kmol)	R^2^
298	− 7.41	35.11	0.14	0.981
303	− 8.39			
308	− 9.19			
313	− 9.86			
318	− 10.47			
323	− 11.02

### Regeneration of corncob cellulose-based hydrogel

**Figure 12:**
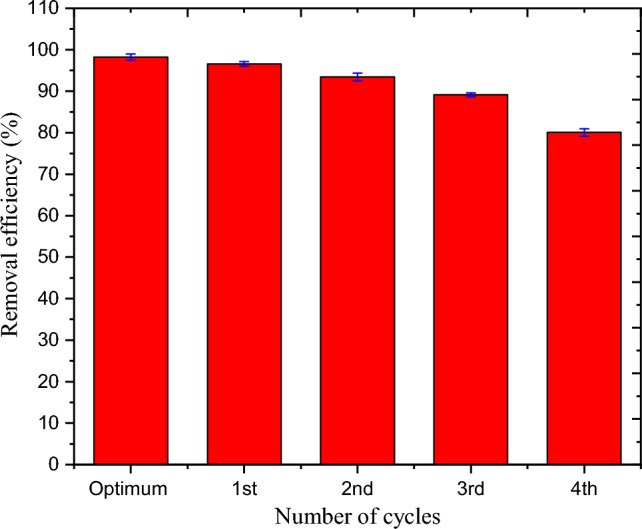
Removal % of MBD by regenerated corncob cellulose based hydrogel.

The most practical way to minimize process costs, recover resources, and minimize the need for producing new adsorbent is to regenerate and reuse adsorbent^46–51^. The hydrogel regeneration process was run through four iterations at optimal process conditions (74.54 mg/L of initial concentration, 2.22 g/L of the dosage, and 80.36 min of contact time at 30 °C). For three hours during each cycle, the adsorbent (10 g of hydrogel) was shaken with 100 mL of MBD solution (100 mg/L). After the adsorption process, the MBD was cleaned with distilled water until it was neutralized for reuse, and then it was washed with a solution of NaOH (0.1 mol/L, 100 mL) for 2.0 h according to the literature^47^. The adsorption efficiency of the hydrogel was investigated for each cycle using a methodology that was similar. The hydrogel's removal efficiency exhibited a tendency to decline over each of the four recycling cycles. Until the third cycle, there was less than a 10% deviation from optimum efficiency. The efficiency decreased from 98.25% to 80.05% at the fourth cycle, with a the18.52% deviation (Fig. 12). This is because MBD ions occupied the active site and porous media of the adsorbent. These findings support the adsorbent's potential for reuse up to the third cycle.

### Comparative study of hydrogel adsorption capacity for methylene blue dye uptake with other adsorbents used in literature

This hydrogel's adsorption capacity was compared to several other literatures. With a maximum uptake of 55.31 mg/g of MBD from contaminated water, the hydrogel adsorbent employed in this investigation has demonstrated the highest capacity. The hydrogel adsorbent effectively reduces MBD from the contaminated water, as shown in Table 8.

**Table 8:** Adsorption capacity comparison of current adsorbet with other adsorbents reported in related literatures.

Adsorbents	Adsorption capacity (mg/g)	References
Activated carbon derived from waste orange and lemon peels	38.00	^48^
Sawdust	7.84	^49^
Torrefied rice husk	6.82	^50^
Micro/nanocomposite of CuMn_2_O_4_/chitosan	54.05	^51^
Rice hull modified by oxalic acid	29.15	^52^
Acid-treated *Cocos nucifera* shell	50.60	^53^
Corncob cellulose-based hydrogel	55.31	This study

### Treatment of the real textile wastewater

To analyze the efficiency of adsorbent on waste water treatment process, the adsorbent has been used in real textile wate water. Before cariyingout adsorption, first textile industry waste water properties (Mg^2+^, Cu^2+^, SO_4_^2**−**^, NO_3_^**−**^, PO_4_^**−**^, and Cl^**−**^) were determined by Uv–vis spectrophotometer. pH of waste water sample was adjusted into neutral. In addition, after treatment, these properties were again determined. The color of real waste water sample was determined using tintometer color units (TCU). The adsorption using cellulose based hydrogel adsorbent was conducted at optimized condition (using 2.22 g/L of dose, 200 rpm at 30 °C for 80.36 min). The maximum removal of MBD in synthetic waste water is 98.25%, whereas in real textile waste water is 64.76%. The reduction in removal efficiency is due to interfering ions in the real waste water as shown in Table 9.

**Table 9:** Physichemical properties of real textile waste water before and after treatment.

Parameters	Before adsorption	After adsorption
Mg^2+^ (mg/L)	56 ± 0.25	3.5 ± 0.07
Cu^2+^ (mg/L)	22 ± 0.23	1.1 ± 0.05
NO_3_^−^ (mg/L)	38 ± 0.25	10 ± 0.03
SO_4_^2−^ (mg/L)	101 ± 0.07	40 ± 0.08
PO_4_^−^ (mg/L)	0.5 ± 0.04	0.05 ± 0.02
Cl^−^ (mg/L)	20 ± 0.16	0.1 ± 0.02
Color (TCU)	2460 ± 0.73	867 ± 0.54

## Conclusion

Water pollution from the textile industry is one major global environmental problem that many nations face. The organic dyes used in the textile and other industries slow down photosynthesis and causes risks and carcinogenic diseases. The alternative and suitable adsorbent for removing dye is hydrogel made from biomass. SEM, FTIR, and XRD equipment were used in this investigation to characterize the hydrogel adsorbent that was synthesized from corncob cellulose. The dosage, contact time, and initial concentration of the process variables were designed using CCD. A total of 20 experiments were conducted. The highest removal efficiency was attained at a dosage of 2.22 g/L, a contact time of 80.36 min, and an initial concentration of 74.54 mg/L. 98.25% of MBD removal was attained under these conditions through experimental testing on synthetic waste water, whereas in real textile industry waste water is reduced into 64.76%. This is due to computing of involved pollutants in real waste water. There were studies done in thermodynamics, kinetics, and isotherms. The adsorption process is better described by the Langmuir isotherm and the pseudo-second-order kinetic model. The conducted experimental process is endothermic and spontaneous, as indicated by all negative values of (ΔG°), positive values of (ΔH°), and (ΔS°), according to thermodynamic studies. Additionally, the experiment for adsorbent regeneration was carried out. Hence, the synthesized hydrogel adsorbent can be used up to the third cycle. All in all, corncob cellulose-based hydrogel is a suitable adsorbent for the removal of MBD.

## Recommendation

For reproduction of the present experiment and investigation of all important adsorption process variables, it has been recommended for future research that will perform on these behalves (Supplementary Information S1).

### Supplementary Information

Supplementary Information.

## Data Availability

Data generated in this investigation can be obtained from corresponding author on reasonable request.
