# Supplementary material for: Expermental investigation on adsorption of methylene blue dye from waste water using corncob cellulose-based hydrogel
Source: Sci Rep. 2024 Feb 24;14:4540. doi: 10.1038/s41598-024-54511-0 (PMC11322434; doi:10.1038/s41598-024-54511-0)
Supplement: Supplementary file 1 — Supplementary Information. [file 41598_2024_54511_MOESM1_ESM.docx]

Analysis of variance (ANOVA) for the suggested quadratic model

| Response 1: Removal efficiency | | |  |  |  |  |
| --- | --- | --- | --- | --- | --- | --- |
|  |  |  |  |  |  |  |
| Source | Sum of Squares | df | Mean Square | F-value | p-value |  |
| Model | 3489.72 | 9 | 387.75 | 263.54 | < 0.0001 | significant |
| a-Adsorbent dose | 1047.35 | 1 | 1047.35 | 711.86 | < 0.0001 |  |
| B-Contact time | 306.47 | 1 | 306.47 | 208.3 | < 0.0001 |  |
| C-Initial concentration | 827.92 | 1 | 827.92 | 562.72 | < 0.0001 |  |
| AB | 19.53 | 1 | 19.53 | 13.28 | 0.0045 |  |
| BC | 23.12 | 1 | 23.12 | 15.71 | 0.0027 |  |
| BC | 18 | 1 | 18 | 12.23 | 0.0057 |  |
| A² | 302.22 | 1 | 302.22 | 205.41 | < 0.0001 |  |
| B² | 12.99 | 1 | 12.99 | 8.83 | 0.014 |  |
| C² | 76.03 | 1 | 76.03 | 51.68 | < 0.0001 |  |
| Residual | 14.71 | 10 | 1.47 |  |  |  |
| Lack of Fit | 12.16 | 5 | 2.43 | 4.75 | 0.0561 | not significant |
| Pure Error | 2.56 | 5 | 0.5116 |  |  |  |
| Cor Total | 3504.43 | 19 |  |  |  |  |
